# Nutrition and health technology assessment: when two worlds meet

**DOI:** 10.3389/fphar.2015.00232

**Published:** 2015-10-20

**Authors:** Marten J. Poley

**Affiliations:** ^1^Institute for Medical Technology Assessment, Erasmus University Rotterdam, Rotterdam, Netherlands; ^2^Intensive Care and Department of Pediatric Surgery, Sophia Children’s Hospital, Erasmus MC, Rotterdam, Netherlands

**Keywords:** health technology assessment, nutrition, nutrition economics, cost-effectiveness, economic evaluation

## Abstract

There is a growing recognition that nutrition may have a positive impact on public health and that it may reduce medical expenditures. Yet, such claims need to be substantiated by evidence. This evidence could be delivered by health technology assessment (HTA), which can be thought of as the evaluation of technologies for clinical effectiveness, cost-effectiveness, and ethical, legal, and social impacts. The application of HTA to the field of “nutrition interventions” is recent. So far, HTA and nutrition have represented two worlds far apart in many respects. This contribution, roughly, addresses the following issues: is there a need for HTAs in the field of nutrition, what would such HTAs look like, and how can the results coming from these HTAs optimally aid policy making?

In essence, HTAs of nutrition have much of the same basic principles and structure as HTAs of “classical” health care treatments. Nevertheless, there are challenges to rigorous HTAs of nutrition interventions, for various reasons. To mention a few: the evidence base for nutrition interventions is less well developed than that for many health care treatments. Furthermore, it is a matter of debate which outcome measures should be used in HTAs of nutrition. For example, one may argue that nutrition not only has health effects, but also effects that are not captured by traditional health-related quality of life measures (e.g., the pleasure of eating, effects relating to ease of use, or effects on well-being).

HTAs in the field of nutrition may deliver information valuable to a wide range of stakeholders, including consumers/patients, health professionals, hospital administrators, insurers, and decision makers. The results of HTAs are typically used in making treatment guidelines, in informing decisions about reimbursement or about public health campaigns, etc. Yet, it is uncertain how the results of HTAs of nutrition can be used optimally. For example, would it be possible to summarize the results of a HTA in a single ratio (such as costs per quality-adjusted life-year gained) and then to either approve or reject the intervention based on this ratio, compared to a certain threshold? Apart from that, in the field of nutrition, it is typically not about reimbursement of a technology. Related to this, it is important that the message from HTAs of nutrition is brought to a range of stakeholders including the general population and that these HTAs are tailored to the decision-making context.

To conclude, a growing need is felt for HTA-type evaluations of nutrition, which are sparse these days. Little thought has been given to developing an optimal methodology for HTAs of nutrition and to how its results should be integrated into policy making. Further work in these areas would stimulate the development of nutrition interventions that yield a gain in societal welfare. To achieve this, the two worlds of HTA and nutrition need to be brought together.

## Introduction

Over the last decades, the pattern of disease shifted from acute diseases to chronic diseases, which are of long duration and generally slow progression. The main types of chronic diseases, also known as non-communicable diseases (NCDs), are cardiovascular diseases, cancers, chronic respiratory diseases, and diabetes. NCDs account for 38 million deaths each year (World Health Organization, 2014). One of the risk factors of NCDs is an unhealthy diet, next to other behavioral risk factors (such as tobacco use) and metabolic/physiological risk factors.

Due to developments like these, there is a growing recognition of the positive impact that nutrition may have on health. This holds for many diseases. To give one example, it has been calculated that 25% of all cancers could be prevented by the right nutrition, together with sufficient physical activity and a healthy body weight (World Cancer Research Fund and American Institute for Cancer Research, 2007). Accordingly, it has been recognized that diet changes have the potential to reduce medical expenditures by considerable amounts. For example, it has been calculated in the United States that a permanent reduction of 100 kilocalories in daily intake would result in savings of related medical costs of 58 billion dollars annually, as it would eliminate some 70 million cases of obesity or overweight (Dall et al., 2009). In this context, it should be noted that nutrition cannot only be effective in the long-term prevention of diseases. It may also have a role in the management of specific disease areas. For example, food supplements or dietary modifications may lead to cognitive improvement (or at least a delayed decline) in patients with early Alzheimer’s disease (Shah, 2013; Swaminathan and Jicha, 2014). To give another example, probiotics have been demonstrated to be beneficial in preventing acute upper respiratory tract infections and in reducing the duration of illness episodes (King et al., 2014; Hao et al., 2015). Finally, nutrition interventions may also have positive effects in the short term: for example, oral nutritional supplements in malnourished hospitalized patients have repeatedly been shown to be associated with a reduced length of hospital stay and thus cost savings (Freijer et al., 2014; Lakdawalla et al., 2014; Walzer et al., 2014).

So, on the whole, it seems that nutrition can have many positive societal effects, in terms of public health and health care costs. Yet, however encouraging this observation is, such claims need to be substantiated by evidence on its positive outcomes, in relation to the additional cost (or: savings) that it may bring about. A discipline that seems well suited to deliver this kind of evidence is called health technology assessment (HTA). HTA has developed strongly over the last decades. From the beginning its intent was to consider the social impact of medical technologies, in order to enable optimal decisions regarding the use and reimbursement of such technologies. HTA has been described as the evaluation of high-priority technologies for efficacy, safety, cost-effectiveness, and current and potential economic, ethical, legal, and social impacts (Perry and Eliastam, 1981). So, HTA takes a multidisciplinary approach, yet assessing the economic impact of health technologies—that is, the balance between costs and (health) benefits—has prevailed in practice. In this respect, the term nutrition economics (as a novel branch of health economics) has become increasingly popular in the field of nutrition (Lenoir-Wijnkoop et al., 2011, 2012; Koponen et al., 2012). In the context of this paper, the term HTA will be used, as it intends to take a broad look at the assessment of nutrition interventions, extending beyond economic impacts alone. But also here, the focus will be on the economic evaluation of nutritional interventions.

Information derived from HTAs has played a growing role in health care decision-making. HTA offers a source of information needed by, for example, policymakers in formulating regulations, by industry in developing products, by health professionals in treating and serving patients, and by consumers in making personal health decisions. It is in line with current trends of evidence-based medicine, where the evidence is not limited to evidence on “clinical” effectiveness only, and growing (financial and other) constraints on the health care sector. To be funded from public funds, interventions increasingly need to have demonstrated safety, effectiveness, and value for money.

The application of HTA to the field of nutrition is recent. That is, from its origin, HTA has mainly been applied to “classical” health care treatments, especially to pharmaceutical interventions and high-technology, expensive treatments such as organ transplantation, kidney dialysis, et cetera. Recently, the conviction has been growing that HTA should also be applied to “nutrition interventions.” Early examples of HTAs in the field of nutrition include HTAs of home parenteral nutrition and HTAs of nutrition interventions targeting disease-related malnutrition. However, nutrition does not parallel health care treatments. Consider that, as will be further explained below, there are many differences for example regarding the “technology” itself, the target population, the policy context, and so on. Consequently, HTAs of nutrition are not necessarily identical to HTAs of health care treatments.

To shed light on some of the questions surrounding the application of HTA to the field of nutrition, this contribution will address the following main questions:

- Is there a need for HTAs in the field of nutrition? Can nutrition interventions be regarded as “health technologies”?
- What would a HTA in the field of nutrition look like, compared to HTAs of “classical” health care interventions?
- Does a HTA in the field of nutrition require other outcome measures, compared to HTAs of “classical” health care interventions? Would the concept of quality of life be a suitable outcome measure?
- How can the results coming from these HTAs optimally aid policy making?

## The Need for HTAs in the Field of Nutrition

Like mentioned above, HTAs have typically focused on “classical” health care treatments, especially on pharmaceuticals and high-technology, expensive treatments. That is, technologies that are used in patients with a diagnosis, that are prescribed by a doctor, and frequently have a direct, observable effect. Nutrition interventions clearly need to be distinguished from health care treatments, for several reasons. They have a different working mechanism: as noted by de Vos et al. (2006), whereas drugs generally contain a single effective component that is specific and only has one target site in the body (producing a large effect), foods consist of a variety of components and can have effects on multiple targets (usually small effects). Furthermore, foods often sort an effect on the long-term, and finally, they are used (mostly on the individual’s own initiative) not only for health purposes, but for other reasons as well (i.e., as a means to stay alive, to satisfy hunger, for pleasure). In addition, foods are consumed every day throughout life, whereas health care treatments are usually required for a short period of time. Finally, to be successful, nutrition interventions often require a change of habits. All this is further complicated by the fact that nutrition covers a wide range of food categories: from conventional food, to functional food, to infant food, to (enteral and parenteral) medical nutrition. So, at least they should be seen as diverse and atypical health technologies.

Nevertheless, there is a need for HTAs in the field of nutrition. Classical HTAs originated from the need that was felt to support decisions regarding the introduction of a technology to the market, reimbursement from the collective insurance, adoption in treatment guidelines, etc. HTAs in nutrition may deliver information that may be valuable to consumers/patients (who have to decide on buying a food product, the costs of which they have to bear themselves), to health professionals (who decide on prescribing of medical nutrition for example), to insurers (who may consider to reimburse functional foods for example) and to decision makers (who are responsible for investing in public educational campaigns and for regulating processes related to food labeling and health claims). This shows that HTA in nutrition is a broad field, which is also shown by the diversity of the subjects covered. These may range from studies of micronutrient deficiencies and malnutrition, to studies of dietary improvements, to studies of functional foods (Gyles et al., 2012). To use another categorization showing the variety of the area of interest: HTAs in nutrition may study subjects as diverse as interventions at the individual level (e.g., advice on diet), interventions at the group level (e.g., group education in schools), and interventions at the population level (e.g., taxes on unhealthy foods).

## HTAs of Nutrition

Having said this, one may wonder what a HTA in nutrition would look like, compared to HTAs of “classical” health care interventions. Is there a need to use adapted techniques, or would regular HTA methods, as they have been developed and refined over the last decades, suffice?

As it will be argued below, in essence, HTAs of nutrition have much of the same basic principles and structure as HTAs of health care, just like the main purpose of those HTAs is the same (i.e., to inform technology-related policy making in health care, against the background of scarce resources). In each field of interest, HTA will basically follow the same steps. Consider for example the 10 basic steps of HTA set out by Goodman and Ahn (1999): (1) Identify assessment topics; (2) Specify the assessment problem; (3) Determine locus of assessment; (4) Retrieve evidence; (5) Collect new primary data (as appropriate); (6) Interpret evidence; (7) Synthesize/consolidate evidence; (8) Formulate findings and recommendations; (9) Disseminate findings and recommendations; (10) Monitor impact.

Nevertheless, there are challenges to rigorous HTAs of nutrition interventions, for various reasons. To mention a few: assessment of nutrition interventions has traditionally mainly relied on non-experimental methods, such as cohort studies, case-control studies, or surveys, as randomized controlled trials (RCTs) are ill-suited for the evaluation of nutrition (Heaney, 2008). So, its evidence base is less well developed than that for many health care treatments, lacking high-quality evidence of cause and effect. Furthermore, also given the absence of RCTs, confounding factors may be present in HTAs of nutrition. This is because people may change their behaviors in other ways as well (as part of an overall lifestyle change), apart from just starting to consume a food product. Next, there is less practical HTA experience in nutrition than in other technologies, such as drugs. Finally, it is a matter of debate which outcome measures can and should be used in HTAs of nutrition, which is the focus of the next section.

So, to conclude, there are specific demands to HTAs of nutrition, which may require such HTAs to move away from traditional HTA methods. Importantly, however, this does not only apply to nutrition, but to other areas as well: for example, the evaluation of public health interventions and long-term (palliative) care services may also raise additional methodological challenges, as has been noted in the literature (Gomes et al., 2009; Weatherly et al., 2009). HTAs of nutrition interventions can benefit from developments in these other areas (and *vice versa*), given that the methodological issues partly coincide with each other.

## Outcome Measures used in HTAs of Nutrition

One of the questions is whether HTAs in the field of nutrition require other outcome measures, compared to HTAs of “classical” health care interventions? In the field of HTA, outcome measures have been developed, to capture the effects of health technologies on patients’ length of life (mortality) and on quality of life. Especially, a large interest has been dedicated to health-related quality of life (HRQoL). HRQoL has been defined as “the physical, psychological, and social domains of health, seen as distinct areas (or domains) that are influenced by a person’s experiences, beliefs, expectations, and perceptions” (Testa and Simonson, 1996). So, HRQoL is a multidimensional construct, encompassing physical, emotional, and social domains. Furthermore, it is about the subjective perception of the relevance and importance of health states to the individual.

Would the concept of HRQoL be a suitable outcome measure in nutrition? To answer this question, a number of observations are worth noting. First, nutrition has effects that are indeed relevant to the patient and that may be captured by measures of HRQoL. Quality-adjusted life-years (QALY), a common outcome measure in HTA, can be used. This has been done, for example, in studies on medical nutrition (Freijer et al., 2014) and in studies on public health interventions, aimed at decreasing fat or sodium intake (Smith-Spangler et al., 2010; Bos et al., 2011). This leaves unaffected that, in the design of a HTA study, measures of HRQoL may be combined with other outcome measures, such as biological and physiological variables (i.e., changes in cell, organ and organ system function) and traditional nutritional outcomes such as energy intake, weight gain, and BMI. For example, one may think of the impact of prebiotic and probiotic foods on the improvement of gut flora, which has attracted much interest recently. Furthermore, it must be realized that nutrition may have an effect only over a long time span, which will have to be taken into account when designing and performing HTAs. Yet, this is not only true for nutrition, it does also apply to some (preventive) health care technologies. So, in sum, if a nutrition intervention aims to increase health, HRQoL measures such as the QALY may be perfectly suitable. However, nutrition may have effects that are not captured by traditional HRQoL measures. We may think of taste, the pleasure of eating, effects relating to comfort or ease of use, effects on well-being or happiness, or intermediate effects on health such as a quicker recovery from surgery or quicker recuperation time needed before receiving a next treatment. This may give nutrition interventions a comparative advantage, ceteris paribus, relative to health care treatments. For example, it may be more pleasurable to eat margarine, yogurt, etc. than to take a drug, just like people may prefer taking a drug to surgery. If an intervention also aims to bring such other “broader” positive effects (other than HRQoL), appropriate outcome measures must be sought to capture the full benefits of nutrition.

Obviously, people attach importance to the health outcomes of health care treatments (and health systems). Yet, there is evidence now that people also care about the processes that precede health outcomes, irrespective of whether they affect health. This has been labeled “process utility” (i.e., utility derived from processes; Donaldson and Shackley, 1997; Ratcliffe and Buxton, 1999; Brennan and Dixon, 2013). Characteristics relating to the process of care may include factors such as invasiveness of the treatment, location and context of the treatment provided, continuity of staff, being treated with dignity, and level of autonomy experienced by the patient. To achieve optimal resource allocation, HTAs should take into account utility resulting from the process of care, next to health gains. This is especially relevant when the field of interest is so broad as to encompass health care interventions (which may range from curative care to preventative care, to comfort care and palliative care) and nutrition interventions.

Finally, it should be recognized that nutrition can have effects on others than the patient/consumer him or herself, which should be taken into consideration in HTA studies (like it is increasingly done in HTAs of health care treatments). Currently, there is only limited evidence on this. One study, for example, evaluated the impact of gastrostomy tube feeding on the HRQoL of carers of children with cerebral palsy. The study reported a significant improvement in the carers’ HRQoL after insertion of a gastrostomy feeding tube, coupled with a significant reduction in feeding times (Sullivan et al., 2004). A recent RCT in newborns with functional gastrointestinal disorders showed that daily administration of a probiotic decreased the onset of gastrointestinal disorders, reduced daily crying time, and led to overall cost savings, by reducing both health care use and lost parental working days (Indrio et al., 2014). These examples highlight that HTAs should take into account effects on others, as nutrition interventions may alleviate the burden on caregivers, improve their HRQoL, avoid productivity losses, and be associated with cost savings.

## Putting the Evidence of HTAs of Nutrition into Practice

The result of any HTA is an assessment of the value (for money) of a health technology. This may, for example, involve the economic costs and clinical effectiveness of a technology, but also ethical and legal pros and cons, the social impact, etc. The results of HTAs are typically used in making treatment guidelines, in informing decisions about reimbursement (in- or exclusion from the benefits package) or about public health campaigns, etc.

The results of a conventional HTA may be summarized in a so-called incremental cost-effectiveness ratio (ICER), which expresses the extra costs required to achieve one additional unit of benefit, such as costs per life-year gained or costs per QALY gained. When the results of an HTA are being used in this way, some threshold would be practical beyond which the technology will not be recommended (approved for funding). However, in no single country, an absolute threshold is being used by decision-makers. Yet, in practice, the chance of rejection increases with decreasing cost-effectiveness. For example, in the UK, it seems that technologies costing £40,000 per QALY have a 50% chance of being rejected (75% at £52,000/QALY; 25% at £27,000/QALY; Dakin et al., 2014). Apparently, it is difficult to fix a certain threshold, and apply it uniformly. There has been plenty of discussion about whether the outcomes should be valued differently for different areas of health care, for example life-threatening severe diseases, treatments targeted at children rather than older people, comfort care rather than curative care, rare diseases, diseases that can be attributed to the responsibility and choice of the individual, etc. (van de Wetering et al., 2014). This suggests that other arguments, most notably equity, play a role in decisions about recommending for or against a technology. Even within health care, we as a society seem to be willing to pay more (i.e., accept a higher threshold) for certain technologies (and disease areas) than for others. What about interventions in the field of nutrition? This is far from clear yet, but likely is related to the characteristics of the beneficiaries.

Furthermore, and perhaps more importantly, it may be reminded here that in the field of nutrition, it is typically not about reimbursement of a technology. Although there have been examples, food products are typically not reimbursed by a third-party payer, but instead paid for out-of-pocket by consumers. Related to this, especially in the area of nutrition, it is important that the message from HTA is brought to the general population (rather than mainly to health care providers or decision-makers). The public could be stimulated to make good food choices, as such, putting the results from HTA into practice. This may be challenging, because consumer food choices are affected by many different factors, including nutrition knowledge, sensory preferences, cost, availability and access to stores, cultural background, social environment, and food marketing (Larson and Story, 2009). This is quite different from health care settings, where, roughly speaking, patients follow their doctor-prescribed regimen (even though the issue of non-compliance must not be underestimated). HTAs in nutrition should ideally take this into account. As a general note, it is worth mentioning here that HTAs of nutrition should target the information needs of different stakeholders, which may include both policy makers, hospital administrators, and the general public. Any HTA of nutrition should think about the following questions: who is the decision maker that needs to informed, which information is most relevant to this decision maker, and what is the decision making context?

## Conclusion

This paper focused attention on HTA and nutrition, two worlds far apart in many respects so far. Increasingly, the need is felt for HTA-type evaluations of nutrition, which provide information relevant to a wide range of stakeholders, including consumers/patients, health professionals, hospital administrators, insurers, and decision makers. Yet, currently, such evaluations, delivering evidence on the social impact of nutrition, are sparse. Moreover, little thought has been given to developing an optimal methodology for HTAs of nutrition, that is, whether current HTA methods suffice or would need adaptation to capture the specific nature of nutrition. Then, a better understanding is required of how to optimally orient HTAs of nutrition to the needs of policy makers and how to integrate the results of these HTAs into policy making (e.g., what criteria can be used to decide for or against a certain nutrition intervention and where to draw the line between cost-effective and cost-ineffective nutrition interventions). Further work in all these areas would stimulate the development of nutrition interventions that generate positive effects on society’s health, and that do so at a reasonable additional cost (or are even associated with cost savings). So, there is a potential gain in societal welfare. To achieve this, the two worlds of HTA and nutrition need to be brought together.

### Conflict of Interest Statement

The author declares that the research was conducted in the absence of any commercial or financial relationships that could be construed as a potential conflict of interest.
